# The Role of p-STAT3 as a Prognostic and Clinicopathological Marker in Colorectal Cancer: A Systematic Review and Meta-Analysis

**DOI:** 10.1371/journal.pone.0160125

**Published:** 2016-08-09

**Authors:** Kun Ji, Mingxuan Zhang, Qi Chu, Yong Gan, Hui Ren, Liyan Zhang, Liwei Wang, Xiaoxiu Li, Wei Wang

**Affiliations:** 1 Department of Pathophysiology, Shenyang Medical College, Shenyang, Liaoning, China; 2 Grade 2012 Clinical Medicine, Shenyang Medical College, Shenyang, Liaoning, China; 3 Department of Social Medicine and Health Management, School of Public Health, Tongji Medical College, Huazhong University of Science and Technology, Wuhan, Hubei, China; 4 Department of Colorectal Surgery, The Second Hospital of Jilin University, Changchun, Jilin, China; 5 Department of health Sciences Research, Mayo Clinic College of Medicine, Rochester, Minnesota, United States of America; 6 Department of Pharmacology, Shenyang Medical College, Shenyang, Liaoning, China; 7 Department of Neurosurgery, The Second Clinical Medical School of Inner Mongolia University for the Nationalities (Inner Mongolia General Forestry Hospital), Yakeshi, Inner Mongolia, China; University of Kentucky, UNITED STATES

## Abstract

**Objective:**

High expression of phosphorylated signal transducer and activator of transcription 3 (p-STAT3) has been detected in a variety of human tumors. However, the association of positive p-STAT3 expression with clinicopathological parameters and the prognosis of colorectal cancer patients remain controversial. To identify the relationship between p-STAT3 expression and clinicopathological parameters and prognosis in patients with colorectal cancer, a systematic review and meta-analysis were performed.

**Methods:**

We performed a comprehensive literature search from PubMed, EMBASE, and SinoMed through 27 March, 2016. Hazard ratios (HRs) with 95% confidence intervals (CI) were combined to evaluate the association between p-STAT3 expression and overall survival of colorectal cancer patients. Odds ratios (ORs) with 95% CI were combined to evaluate the association between p-STAT3 expression and clinicopathological parameters in patients with colorectal cancer.

**Results:**

Seventeen studies including a total of 2,346 colorectal cancer patients were included in this meta-analysis. The combined HR was 1.43 (95% CI: 1.23–1.67, P < 0.001), which suggested a positive relationship between p-STAT3 overexpression and poorer overall survival of colorectal cancer patients. In addition, the results indicated that positive p-STAT3 expression was significantly associated with the presence of lymph node metastasis (OR: 2.43, 95% CI: 1.18–5.01, P = 0.02) but was not associated with TNM stage, tumor differentiation or gender.

**Conclusion:**

The meta-analysis results suggest that p-STAT3 overexpression is unfavorable for the prognosis of colorectal cancer patients, and p-STAT3 overexpression is associated with the presence of lymph node metastasis among colorectal cancer patients.

## Introduction

Colorectal cancer (CRC) represents close to 10% of global cancer incidence with an estimated 746 000 new cases in men and 614 000 new cases in women, and it was the fourth most common cause of death from cancer with an estimated 694 000 deaths worldwide in 2012. The estimated new cases mainly occurred in Europe (32.9%), East and Central Asia (19.3%), and China (18.6%). The estimated deaths mostly occurred in Europe (31.0%), China (20.1%), and East and Central Asia (18.5%). The 5-year relative survival of colorectal cancer ranged from 65.5% (diagnosis in 1999–2003) to 66.1% (diagnosis in 2004–2010) [1]. The incidence rates are substantially higher in men than women, and the mortality rates are lower in women than men. Diet, lifestyle, medications, smoking, obesity, and sedentary lifestyle were associated with an increased risk of colorectal cancer [2–3]. Moreover, genetic changes may play a crucial role in the pathogenesis of colorectal cancer.

Signal transducer and activator of transcription (STAT) is a member of the family of transcription factors that regulates cell proliferation, cellular transformation, tumor formation and immune responses and is also involved in tumorigenesis, metastasis and angiogenesis [4]. To date, seven members of the STAT family have been identified including STAT1, STAT2, STAT3, STAT4, STAT5a, STAT5b and STAT6 [5]. Signal transducer and activator of transcription 3 (STAT3) is a type of latent cytoplasmic signaling molecule and it has two subtypes: full-length STAT3a and truncated STAT3β. STAT3a is the more commonly expressed subtype in cells, and STAT3β can inhibit the transactivation function of STAT3a [6]. Under the action of cytokines and growth factors, STAT3 transmits signals from the cell surface into the nucleus while STAT3 is phosphorylated, and this process requires the participation of receptor-associated kinases [7–8]. STAT3 is mainly activated by tyrosine phosphorylation at position 705, followed by its dimerization, nuclear translocation, DNA binding, and target gene transcription. In normal cells, STAT3 activation is strictly controlled, but activated STAT3 has been detected in a variety of human tumor specimens and tumor cell lines, which suggests it plays a critical role in the occurrence and development of tumors [9–11].

Recent studies have demonstrated that increased p-STAT3 expression was detected in patients with colorectal carcinoma. However, the prognostic value and clinicopathological parameters of p-STAT3 expression in colorectal cancer were undefined. Therefore, a systematic review and meta-analysis were conducted to explore the relationship between p-STAT3 expression and clinicopathological parameters, as well as the overall survival of colorectal cancer patients.

## Materials and Methods

### Search strategy

We conducted a systematic literature search to identify all relevant studies using electronic databases, including PubMed, EMBASE, and Sinomed (the ending date of our search was March 27, 2016). The following keywords were used: (intestine OR colorectal OR colon OR rectum) AND (tumor OR neoplasm OR carcinoma OR cancer) AND (STAT3 Transcription Factor OR Signal transducer and activator of transcription 3 OR STAT3). Reference lists from the included studies were also manually reviewed to identify potentially relevant studies.

### Inclusion criteria

All eligible studies were required to assess the relationship between p-STAT3 overexpression and clinicopathological parameters or overall survival of colorectal cancer patients. Hence, we excluded some studies that had no correlations or had low correlations by reading titles and abstracts. Subsequently, the eligible criteria were further narrowed down, and studies were included if they met all of the following inclusion criteria: (1) studies used an immunohistochemical method to detect the expression of p-STAT3, (2) the sample of studies was from clinical colorectal cancer patients, (3) the association between p-STAT3 overexpression and overall survival was assessed by hazard ratio (HR) with 95% confidence intervals (CI) or could be calculated from the published data, (4) the study provided sufficient data to calculate an odds ratio (OR) and then evaluate the association between p-STAT3 overexpression and clinicopathological parameters, such as gender, tumor differentiation, lymph node metastasis or TNM stages, (5) if the study was published with the similar data in more than one journal, only the recent or the most complete study was included and (6) all studies was published in Chinese and English should be from the Chinese core journals. Two authors (MXZ and QC) preformed the search and identification independently.

### Data extraction

Two authors (KJ and YG) used a unified form to extract relevant information independently from all of the included studies, which included the name of the first author, publication year, journal, the language of the publication, the source of the samples, country, age, gender, information about treatment, type of cancer, the numbers of patients, the location of p-STAT3 expression, scoring method, clinicopathological parameters (including lymph node metastasis, TNM stage, and tumor differentiation), follow-up time, cut-off value, antibody used for evaluation, and HR with corresponding 95% CI. In addition, disagreements (controversial problems) were resolved by discussion with a third author (LYZ).

### Quality assessment

Methodological quality was independently assessed according to the quality scale for biological prognostic factors from the European Lung Cancer Working Party (ELCWP) by two authors (XXL and HR) [12]. Briefly, the ELCWP scale was founded on four categories: (1) scientific design, (2) laboratory methodology, (3) generalizability and (4) result analysis. Each of these categories can be awarded a maximum score of 10 points, and the overall score is 40 points. Two authors calculated the scores independently, and a consensus score was achieved. The final score of each study was expressed as a percentage, ranging from 0 to 100% higher scores indicated a better methodological quality. The category “results analysis” only evaluated the studies that included the survival data thus the studies that only compared the association between p-STAT3 overexpression and clinicopathological parameters would receive a lower score in this category.

### Statistical analysis

HR and 95% CI were used to estimate the relationship between p-STAT3 overexpression and overall survival in colorectal cancer patients. If HR and 95% CI were given in the studies, we used them directly. If the study only provided Kaplan-Meier survival curves, we extracted the data using the software Engauge Digitizer version 4.1 (http://sourceforge.net/projects/digitizer) and calculated the HR with 95% CI using the program files supplied by Jayne F Tierney [13] (http://www.biomedcentral.com/content/supplementary/1745-6215-8-16-S1.xls). ORs with 95% CI were used to evaluate the correlation between p-STAT3 overexpression and clinicopathological parameters in colorectal cancer patients, including gender (female versus male), lymph node metastasis (present versus absent), TNM stage (III-IV versus I-II), and tumor differentiation (poorly versus well-moderately).

In this meta-analysis, OR and HR with 95% CI were pooled by a random effects model, and heterogeneity was evaluated using the I^2^ statistic [14]. If I^2^ > 50%, heterogeneity was regarded as significant among the studies, and we conducted subgroup analysis and meta-regression analysis to explore the source of the heterogeneity, including the ethnicity of patients (Asia versus non-Asia) and the location of p-STAT3 expression (nucleus versus cytoplasm and nucleus). To explore the stability of our meta-analysis, we also performed sensitivity analysis by omitting individual studies and comparing different effect models (fixed effects model and random effects model). Meanwhile, if the number of included studies was 10 or more, funnel plots, the Egger test and the Begg test were used to assess the potential publication bias. If publication bias was detected, the trim and fill method was used to assess or adjust for the presence of publication bias. Statistical analyses were performed using Review Manager 5 (version: 5.2, Cochrane Informatics and Knowledge Management Department, http://tech.cochrane.org/revman/download) and STATA (version 12.0, StataCorp, College Station, TX). Additionally, to evaluate the result of the quality assessment, the Mann-Whitney U test was conducted using the software Statistical Product and Service Solutions 14.0 (SPSS Inc., Chicago, IL). P-values were two-tailed and statistical significance was set at 0.05.

## Results

### Study selection

**Fig 1:**
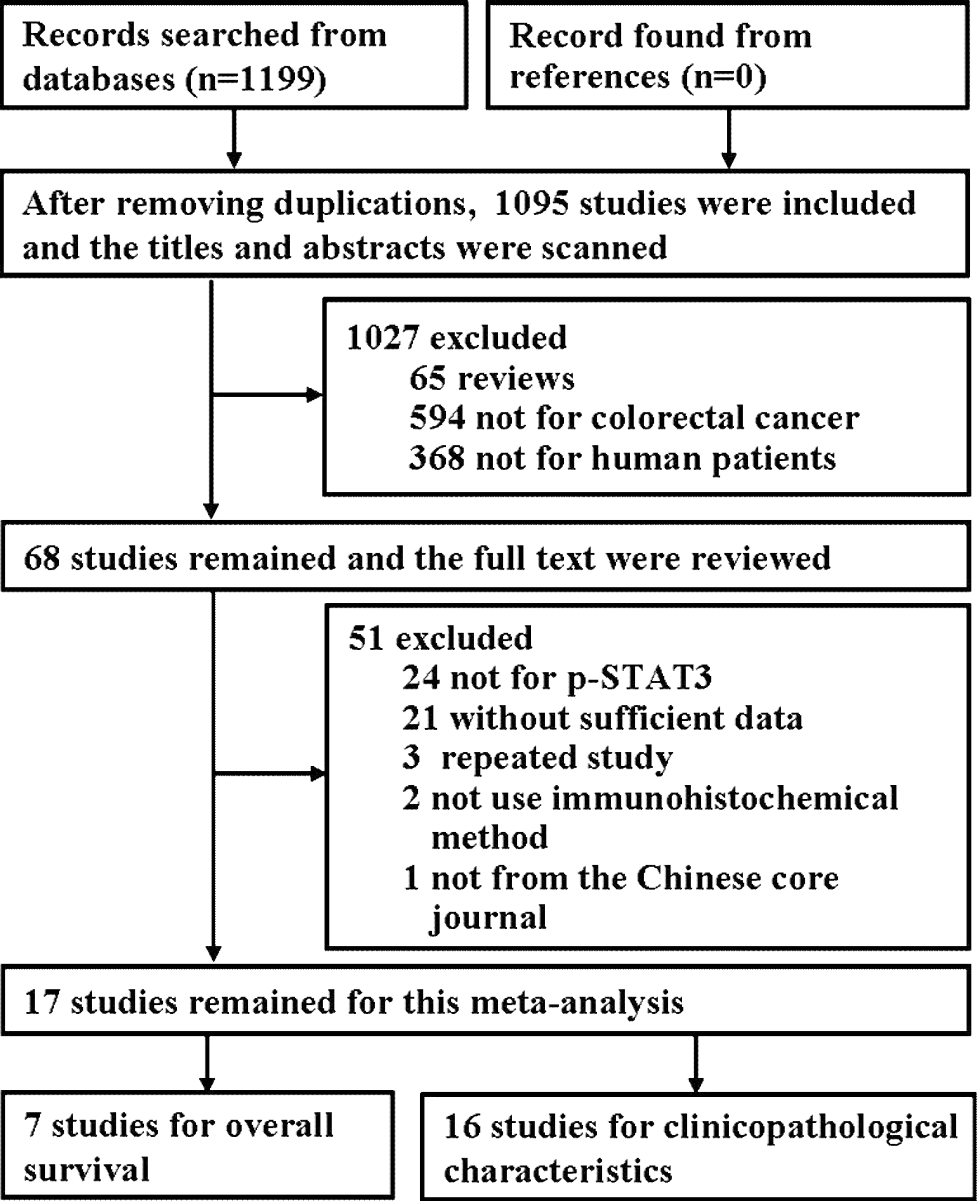
Flow diagram of the included studies.

Initially, 1,199 relevant studies were identified by electronic database searches, of which 662 studies were from PubMed, 433 studies were from Embase and 104 studies were from SinoMed (Fig 1). After removing duplicates, 1,095 studies remained. By screening titles and abstracts, 1,027 studies were excluded that were not relevant based on the inclusion criteria. After reading the full text of 68 studies, 51 studies were excluded. Among these 51 studies, 24 studies reported the association of STAT3 expression with clinicopathological parameters or poorer overall survival of colorectal cancer, 21 studies did not supply sufficient data, 3 studies were published delicately, 2 studies did not use immunohistochemical methods to detect the expression of p-STAT3 and 1 study was not from the Chinese core journals. Finally, the remaining 17 studies were included in the meta-analysis [15–31].

### Study characteristics

The major characteristics of these 17 studies are shown in Table 1, including 12 Asian studies [15,17–19,23–25,27–29,31,30], 3 European studies [16,20–21], 1 African study [26], and 1 United States study [22]. The total number of sample sizes ranged from 22 to 724 with a total of 2346. The studies were published between 2005 and 2016. In these 17 studies, 2 studies reported colon cancer [18,30], 1 study reported rectal cancer [16], and the remaining studies reported on colorectal cancer. Lymph node metastasis was recorded in 10 studies [15–16,19–20,23,25–27,29,31], TNM stage was reported in 10 studies [15,18–19,21–24,26,28–29], tumor differentiation was involved in 12 studies [15,25,27,16,22–23,26,17,19–20,29,31], and HRs and 95% CIs were extracted from 7 studies [21,24,15–16,22,29–30]. Concerning the location of p-STAT3 expression, 4 studies noted the cytoplasm and nucleus [15,25,23,20], 12 studies noted the nucleus [21,24,18,27,16,22,26,28,17,19,29,31], and in 1 study the location was not mentioned [30]. The gender of the patients was not mentioned in 4 studies [18,23,26,17] and the age of patients was not reported in 6 studies [27,22–23,28,17,29]. An immunohistochemiscal method was used to evaluate the expression of p-STAT3 in all of the included studies. A rabbit polyclonal antibody was used in 5 studies [21,16,22,19–20], a goat polyclonal antibody was used in 3 studies [15,25,23], and definitive antibody information was not mentioned in 9 studies [24,18,27,26,28,17,29,31,30]. The scoring method of p-STAT3 expression was different among the studies. The percentage of positive cells was used in 8 studies [21,24,15,25,16,23,26,17], both staining intensity and percentage of positive cells were used in 7 studies [27–28,19–20,29,31,30], and the scoring method was not mentioned in 2 studies [18,22]. The median percentage of positive p-STAT3 expression was 61.98%.

**Table 1:** Charateristics and results for 17 included studies.

Author	Publication year	Country	Age (years)	Gender (M/F)	Cancer type	Location	NO.	Positive (%)	Stage	LN	Grade	Anti-body	Scoring method	Cut-off value	Follow-up (months)	HR estimate	Quality Score (%)
Kusaba[15]	2006	Japan	median 65.6 (44–86)	66/42	colorectal	Nu and Cyt	108	57.41	I- II:70 III-IV:38	34	I- II:100 III:4	G pAb	P	>15%	median 43.7 (0.71–60)	K-M	75.54
Monnien[16]	2010	France	median 66 (37–80)	76/28	rectal	Nu	104	37.50	III-IV:104	31	I- II:97 III:1	R pAb	P	>15%	6–60	K-M	83.45
Xiong[17]	2008	China	_	_	colorectal	Nu	38	100.00	_	_	I- II:33 III:3	pAb	P	>15%	_	_	44.76
Lin[18]	2005	Japan	(54–74)	_	colon	Nu	22	68.18	II:11 IV:11	_	_	pAb	_	_	_	_	43.36
Zhong[19]	2014	China	median 62 (40–76)	29/21	colorectal	Nu	50	76.00	I- II:22 III-IV:28	30	I- II:36 III:14	R pAb	SI, P	>1	_	_	41.37
Zizi-Sempet Zoglou[20]	2012	Greece	mean 65±2.5	85/50	colorectal	Nu and Cyt	135	62.96	_	79	I- II:106 III:18	R pAb	SI, P	≥16%	_	_	43.33
Dobi[21]	2013	France	median 65.5 (29–91)	50/44	colorectal	Nu	94	24.50	II:19 III-IV:75	_	I:34 II-III:56	R pAb	P	>15%	median 22	K-M	60.71
Morikawa [22]	2011	America	_	266/458	colorectal	Nu	724	51.80	I- II:384 III-IV:301	_	I:61 III:660	R pAb	_	_	median 129	K-M	76.19
Park[23]	2008	Korea	_	_	colorectal	Nu and Cyt	174	77.59	I- II:126 III:48	65	I- II:146 III:12	G pAb	P	>15%	_	_	54.82
Kawada[24]	2006	Japan	mean 66.4 (19–82)	57/33	colorectal	Nu	90	44.44	I- II:45 III-IV:45	_	_	pAb	P	>30%	2–57	K-M	51.82
Kusaba[25]	2005	Japan	median 65.6 (32–87)	52/43	colorectal	Nu and Cyt	95	72.63	_	33	I- II:86 III:3	G pAb	P	>15%	_	_	48.69
Shareef[26]	2009	Egypt	mean 45±17.2 (21–76)	_	colorectal	Nu	45	82.22	I- II:8 III-IV:22	19	I- II:34 III:11	pAb	P	≥15%	_	_	48.27
Lin[27]	2013	China	_	69/60	colorectal	Nu	129	60.47	_	32	I- II:99 III:30	pAb	SI, P	>3	_	_	43.04
Xiong[28]	2012	China	_	11//21	colorectal	Nu	35	65.71	I- II:16 III-IV:16	_	_	pAb	SI, P	>3	_	_	44.82
Fan[29]	2015	Taiwan		158/84	colorectal	Nu	243	61.00	I- II:84 III-IV:158	55	I- II:30 III:215	Ab	SI, P	>10	0–144	HR	55.95
Ren[30]	2016	China	median 65 (26–90)	90/75	colon	_	165		I- II:93 III-IV:72	67	_	Ab	SI, P	≥6	_	HR	56.67
Li[31]	2015	China	median 66 (33–85)	52/43	colorectal	Nu	95	50.52	I- II:86 III-IV:9	43	I- II:79 III:16	Ab	SI, P	>4	_	_	41.55

M, male; F, female; Nu, nucleus; Cyt, cytoplasm; N, patients number; LN, Lymph node metastasis; SI, staining intensity; P, percentage of positive cells; R, rabbit; G, goat; pAb, polyclonal antibody; K-M, Kaplan-Meier survival curves; “-“, not mentioned.

### Quality assessment

Quality assessment for each study was conducted by evaluating study design, laboratory methodology, generalizability, results analysis, and calculating a global quality score. The median global quality score of the included studies was 48.69% (range 41.37% to 83.45%) (Table 2). When we compared the global scores of the studies with survival data (n = 7) with those without survival data (n = 10), the median overall quality scores were 60.71% and 44.06%, respectively, and the difference was statistically significant (P = 0.001). Otherwise, when we compared the quality of study design, the studies with survival data had a better score than those without survival data (P = 0.009). There was no statistically significant difference between studies from Asia and non-Asia for the global score (median of 46.76% and 60.71%, P = 0.140).

**Table 2:** Study quality assessment according to the ELCWP Scale.

	Number.of studies	Design*	Laboratory methodology*	Generalizability*	Results analysis*	Global Score (%)
All Studies	17	8	5.214	4.333	0	48.69
Survival Data	7	9	6.286	4.333	7.5	60.71
No Survival Data	10	8	5.107	4.333	0	44.06
P-value		0.009	0.186	0.372	0	0.001
Asia	12	8	5.214	4.333	0	46.76
Non-Asia	5	8	6.286	5	5	60.71
P-value		0.265	0.315	0.284	0.193	0.140

Score distributions are summarized by the median values

*, score out of 10.

### Meta-analysis results

#### P-STAT3 expression and overall survival

**Fig 2:**
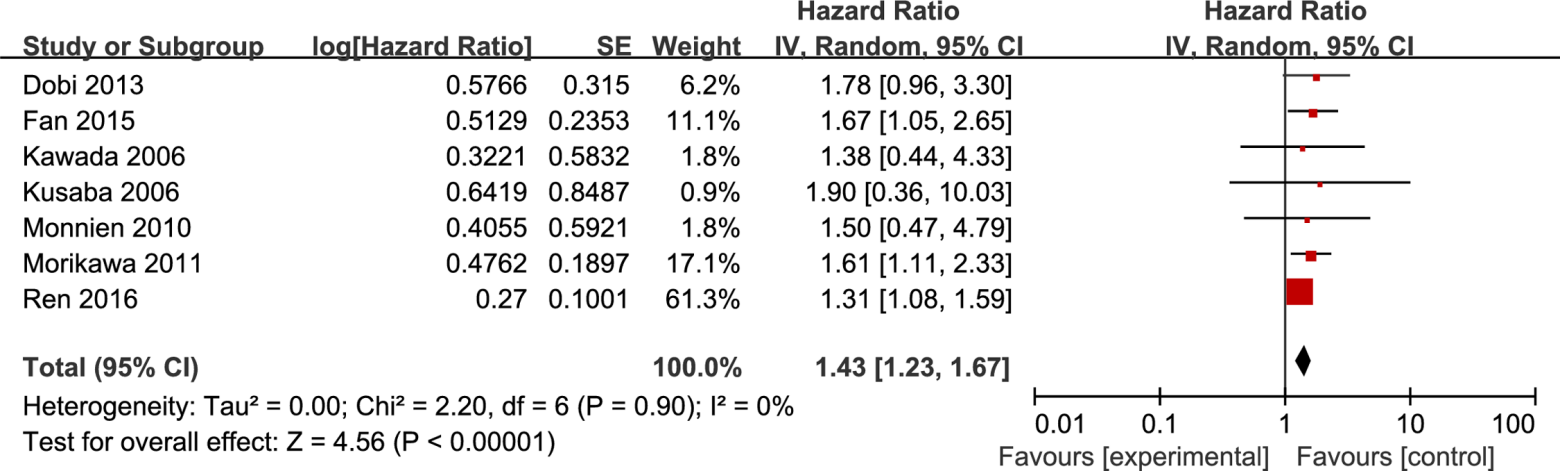
Forest plot for the association of p-STAT3 expression with overall survival in colorectal cancer patients.

Seven studies evaluated the association between p-STAT3 overexpression and overall survival in colorectal cancer patients. As shown in Fig 2, the combined HR was 1.43 (95% CI: 1.23–1.67, P < 0.001, I^2^ = 0%) by random effects model, and when the fixed effects model was used, the same result was obtained, which revealed the positive correlation between p-STAT3 overexpression and poorer overall survival in colorectal cancer patients. Sensitivity analysis was performed by omitting any single study each time in turn, and the result suggested that there was no significant influence on the combined HR (Table 3).

**Table 3:** Sensitivity analysis for p-stat3 expression with overall survival.

Study omitted	HR (95% CI)
Dobi 2013	1.41 (1.20–1.65)
Fan 2015	1.40 (1.19–1.65)
Kawada 2006	1.43 (1.23–1.67)
Kusaba 2006	1.43 (1.22–1.66)
Monnien 2010	1.43 (1.22–1.67)
Morikawa 2011	1.40 (1.18–1.65)
Ren 2016	1.64 (1.28–2.10)
Combined	1.43 (1.23–1.67)

#### P-STAT3 expression and clinicopathological parameters

**Fig 3:**
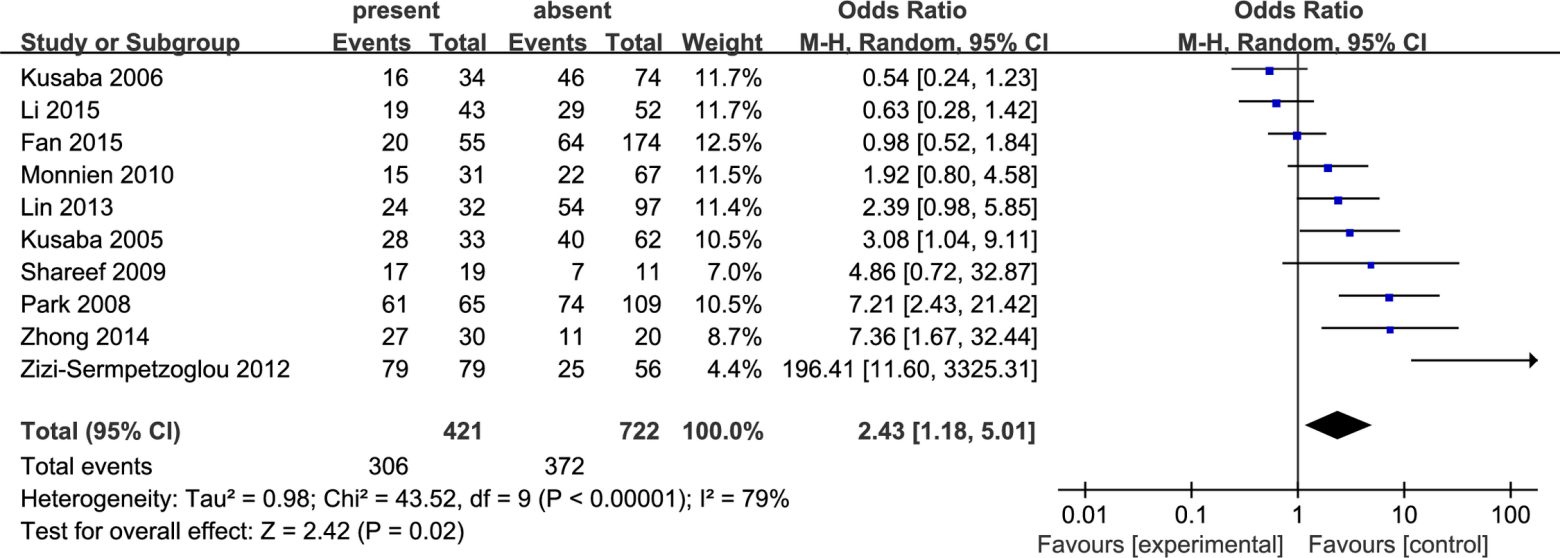
Forest plot for the association of p-STAT3 expression with lymph node metastasis in colorectal cancer patients.

**Fig 4:**
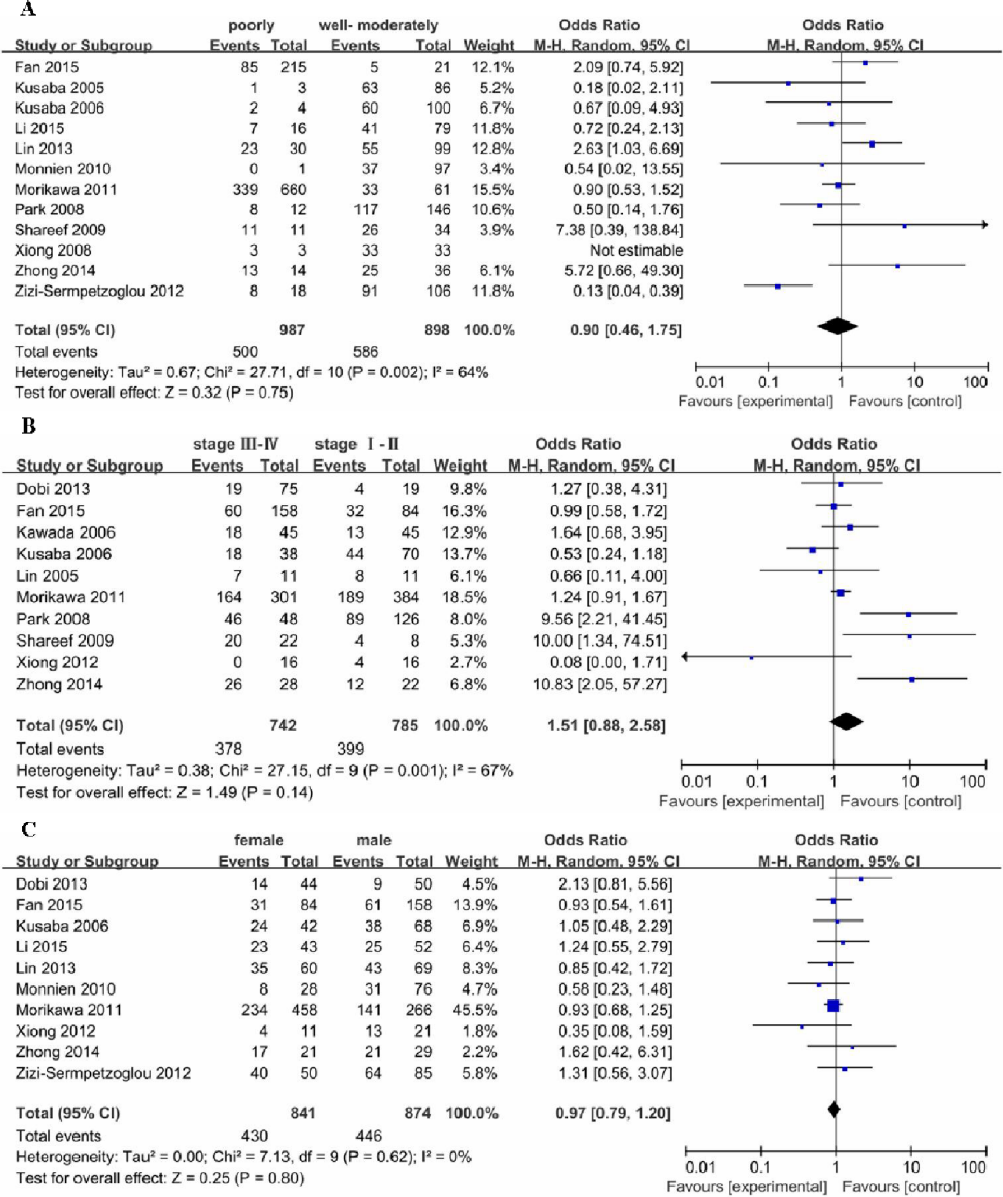
Forest plots of odds ratios (OR). (A) OR for the association of p-STAT3 expression and tumor differentiation in colorectal cancer patients. (B) OR for the association of p-STAT3 expression and TNM stage in colorectal cancer patients. (C) OR for the association of p-STAT3 expression and gender in colorectal cancer patients.

Pooled ORs were used to evaluate the relationship between p-STAT3 overexpression and clinicopathological parameters of colorectal cancer patients by random effects model. The results showed that the presence of lymph node metastasis had a statistically significant association with p-STAT3 overexpression (OR: 2.43, 95% CI: 1.18–5.01, P = 0.02, I^2^ = 79%) (Fig 3); however, p-STAT3 expression was not associated with the other clinicopathological parameters in colorectal cancer patients, including TNM stage (III-IV versus I-II: OR: 1.51, 95% CI: 0.88–2.58, P = 0.14, I^2^ = 67%), tumor differentiation (poorly versus well-moderately OR: 0.90, 95% CI: 0.46–1.75, P = 0.75, I^2^ = 64%), and gender (female versus male OR: 0.97, 95% CI: 0.79–1.20, P = 0.80, I^2^ = 0%) (Fig 4). Compared with the random effects model, when a fixed effects model was applied, the association between p-STAT3 overexpression and TNM stage showed obvious statistical significance (OR: 1.31, 95% CI: 1.05–1.62, P = 0.009, I^2^ = 70%), but the association among tumor differentiation and gender was not significant.

Furthermore, there was a significant heterogeneity in the analysis of the association between p-STAT3 expression and lymph node metastasis in colorectal cancer. Therefore subgroup analysis and meta-regression were performed based on ethnicity (Asia versus non-Asia) and the location of p-STAT3 expression (nucleus versus cytoplasm and nucleus) (Table 4). When subgroup analysis was performed based on ethnicity, the combined ORs and 95% CI were 1.81 (0.86–3.80) (Asia) and 9.46 (0.66–135.11) (non-Asia), and the meta-regression analysis result showed that no statistically significantly difference was found between the subgroups (P = 0.08). When subgroup analysis was conducted based on the location of p-STAT3 expression, the combined ORs and 95% CI were 1.73 (0.91–3.32) (nucleus) and 4.99 (0.75–33.26) (cytoplasm and nucleus), and no significant difference was found by meta-regression analysis (P = 0.99). In addition, we pooled the OR between p-STAT3 expression and lymph node metastasis by fixed effects model, compared with the random effects model, and no obvious difference was found (OR: 2.11; 95% CI: 1.61–2.76; P <0.001; I^2^ = 79%). Subsequently, sensitivity analysis was conducted by excluding one study at a time, and the results showed that the pooled ORs and heterogeneity did not change statistically (Table 5).

**Table 4:** Subgroup analysis of p-STAT3 expression and clinicopathological feature of colorectal cancer.

Subgroup	Number of studies	Pooled data (random effects model)			Heterogeneity		
		OR	95% CI	P-value	Chi^2^	I^2^	P-value
Ethnicity							
Asia	7	1.81	0.86–3.80	0.12	27.34	78%	0.0001
Non-Asia	3	9.46	0.66–135.11	0.1	13.4	85%	0.001
Location							
Nucleus	6	1.73	0.91–3.32	0.1	13.27	62%	0.02
Nucleus and Cytoplasm	4	4.99	0.75–33.26	0.1	29.63	90%	<0.00001

**Table 5:** Sensitivity analysis for p-STAT3 expression with Lymph node metastasis.

Study omitted	OR (95% CI)
Fan 2015	2.87 (1.24–6.61)
Kusaba 2005	2.41 (1.09–5.33)
Kusaba 2006	2.95 (1.39–6.26)
Li 2015	2.92 (1.35–6.34)
Lin 2013	2.51 (1.11–5.70)
Monnien 2010	2.60 (1.14–5.94)
Park 2008	2.09 (1.01–4.33)
Shareef 2009	2.32 (1.09–4.95)
Zhong 2014	2.18 (1.03–4.59)
Zizi-Sermpetzoglou 2012	1.92 (1.03–3.55)
Combined	2.43 (1.18–5.01)

### Publication bias

**Fig 5:**
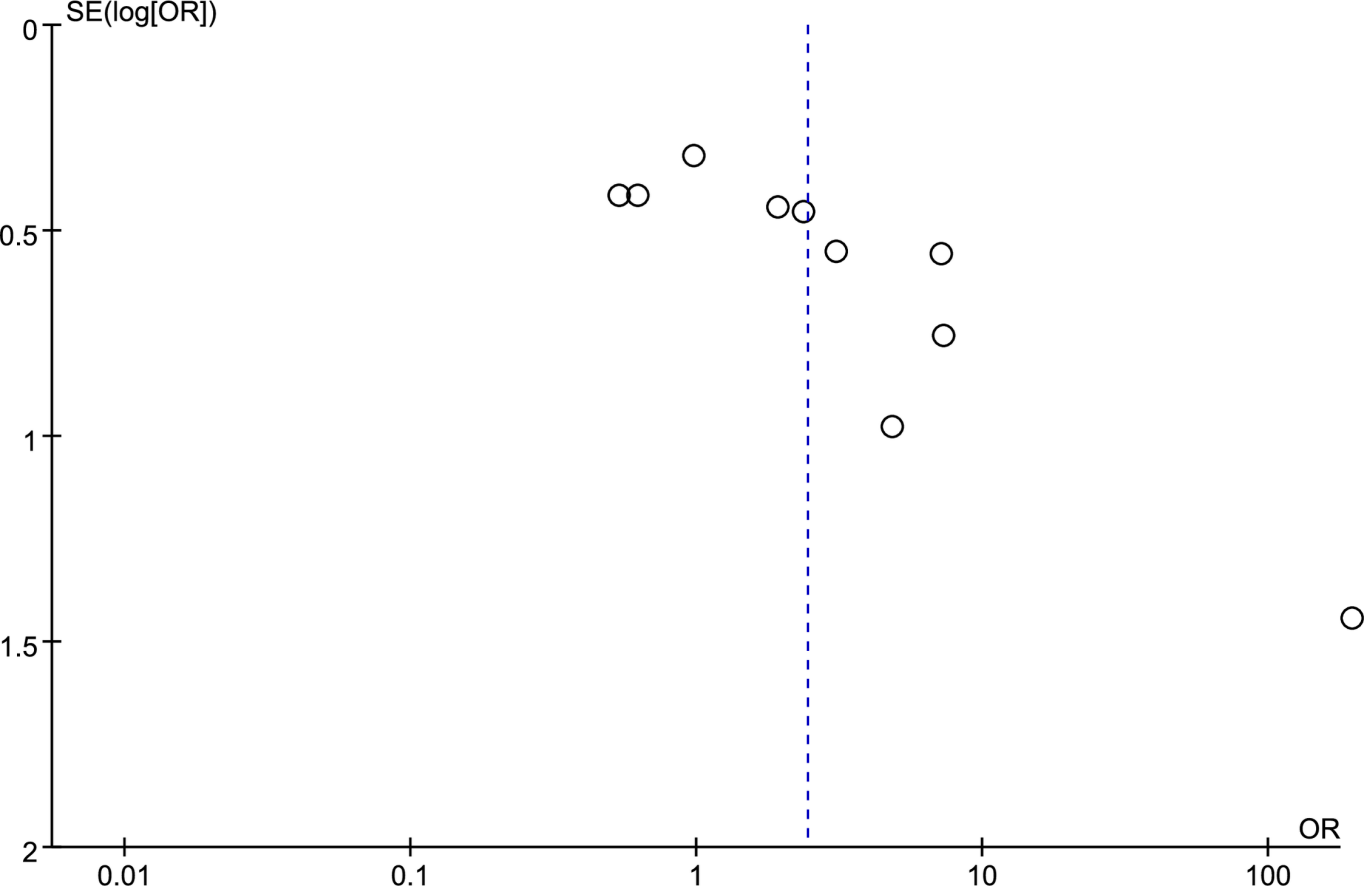
Funnel plot for p-STAT3 expression with lymph node metastasis in colorectal cancer patients.

Tests for funnel plot asymmetry were conducted only when there were at least 10 studies included in the meta-analysis. Thus, the potential publication bias was assessed between p-STAT3 expression and lymph node metastasis by funnel plot, Egger test and Begg test in colorectal cancer. As shown in Fig 5, the shape of the funnel plot showed evidence of asymmetry, and the Egger test and the Begg test indicated that the publication bias was significant (p = 0.003, p = 0.007).

## Discussion

Colorectal cancer was the third of most commonly diagnosed cancer in women and men in 2015 [32]. Over the past few decades, the incidence rate of colorectal cancer had markedly declined from 66.31% (1985) to 39.79% (2011), due to the changes in risk factors and the introduction of colorectal cancer screening, such as the guaiac fecal occult blood test (gFOBT), fecal immunochemical test (FIT), flexible sigmoidoscopy (FS) and colonoscopy[3,33]. Approximately 50% of recently diagnosed colorectal cancer patients will develop metastatic cancer, and the rapid metastasis to the enterocoelia and pelvic cavity is the major challenge for treatment [34]. The 5-year survival rates for patients with distant stages (12.9%) are lower than localized (89.8%) and regional stages (70.5%) in colorectal cancer [1]. In recent years, some molecular markers had been identified that were related to the survival rate of colorectal cancer patients, which may be helpful in the improvement of therapeutic strategies and survival rates of colorectal cancer patients. For example, APC promoter methylation may be a potential biomarker for the carcinogenesis of colorectal cancer [35], SMAD4 expression was a prognostic marker for survival in colorectal cancer patients [36], and the overexpression of CXCR4 reduced overall survival in colorectal cancer patients [37].

In recent evidence from previous studies, STAT3 was constitutively activated in some types of cancer. For example, p-STAT3 expression was detected in patients with gastric cancer [38], esophageal cancer [39], lung cancer [40], renal cell cancer [41], cervical cancer [42], prostate cancer [43], and colorectal cancer [44–45]. Activated STAT3 may play an important role in the development of cancer and phosphorylation was the major mechanism of STAT3 activation. Many studies have investigated the correlation between p-STAT3 and malignant tumors; for example, studies confirmed that high p-STAT3 expression was associated with poor prognosis among patients with non-small-cell lung cancer [46]. Our research has demonstrated that the increased p-STAT3 expression not only predicts poor prognosis of gastric carcinoma patients, but is also associated with worse tumor differentiation and lymph node metastasis in patients with gastric carcinoma [47].

Besides, in previous meta-analysis papers, we searched 2 studies which involved colorectal cancer and p-STAT3. One study reported that p-STAT3 expression was not associated with overall survival which included 12 studies concerning tumors, including 2 studies for breast cancer, 2 for gastric cancer, 2 for NSCLC, 1 for colorectal cancer, 1 for astrocytomas, 1 for esophagealcarcimoma, 1 for hepatocellular carcinoma, 1 for osteosarcoma, and 1 for pharyngeal cancer [48]; the other study reported that elevated p-STAT3 expression was related with inferior overall survival in patients with cancers of the digestive system, including 4 colorectal cancer, 9 gastric cancer, 3 hepatocellular carcinoma, 3 esophagus cancer and 3 pancreatic cancer, and increased expression of p-STAT3 is significantly related with tumor cell differentiation and lymph node metastases [49]. According to the above, these published papers did not explain the association between p-STAT3 and colorectal cancer directly. Thus, it is necessary to investigate the relationship between p-STAT3 and colorectal cancer.

In the present study, 7 studies including 1528 cases were included to evaluate the association between p-STAT3 expression and overall survival in colorectal cancer patients. The combined HR and 95% CI demonstrated that p-STAT3 overexpression was significantly associated with poorer overall survival in colorectal cancer patients. When we conducted a sensitivity analysis, we found that the combined HRs were not changed. Additionally, the relationship of p-STAT3 overexpression with clinicopathological parameters of colorectal cancer was investigated. We found that overexpression of p-STAT3 was significantly associated with the presence of lymph node metastasis in colorectal cancer. In the analysis, we included 421 patients who have the presence of lymph node metastasis and 722 patients who did not have the presence of lymph node metastasis. The p-STAT3 expression in patients with the presence of lymph node metastasis was 2.43 times higher than the patients without the lymph node metastasis.

However, the heterogeneity was observed; therefore, subgroup analysis and meta-regression analysis were conducted to identify the potential sources of heterogeneity based on ethnicity (Asian or non-Asian) and the location of p-STAT3 expression (nucleus versus cytoplasm and nucleus). However, both results showed that the heterogeneity was not significantly influenced by these factors. Subsequently, to further explore the sources of heterogeneity and the stability of the pooled results, sensitivity analysis was performed by excluding one study at a time. We found that there was no significant influence on the stability of the results. The source of heterogeneity may result from certain factors, such as the number of samples, the source and concentration of the antibody, different cut-off values, the p-STAT3 positive expression rate, and the age of the patients. The relationship between the overexpression of p-STAT3 and other clinicopathological parameters (including TNM stage, tumor differentiation, and gender) was not statistically significant by random effects model in colorectal cancer patients. We then used a fixed effects model to investigate the association between p-STAT3 overexpression and clinicopathological parameters, compared with a random effects model, and we found that the association between p-STAT3 overexpression and TNM stage had statistical significance by fixed effects model. This finding indicates that the result was unstable; therefore, we could not determine the relationship between the p-STAT3 overexpression and the TNM stage of patients with colorectal cancer. We constructed a funnel plot and performed an Egger test and a Begg test to assess potential publication bias regarding p-STAT3 expression and lymph node metastasis in colorectal cancer patients. The funnel plot showed the evidence of asymmetry, and the Egger test and the Begg test indicated that publication bias was observed.

However, it should be acknowledged that the meta-analysis in this study had some limitations. Firstly we only included the studies using an immunohistochemical method to detect p-STAT3 expression. Immunohistochemistry is a simple and fast method for detecting molecular markers of cancer, but different primary antibodies and concentrations might produce potential bias. For example, a rabbit polyclonal antibody was used in 5 studies, a goat polyclonal antibody was used in 3 studies, and 9 studies did not report antibody information. Secondly, the scoring method for positive p-STAT3 was different; for example, some studies used the percentage of positive cells and others used both staining intensity and the percentage of positive cells. Thirdly, a potential influence may be related to the approach of HR extraction. In all of the studies evaluating the relationship between p-STAT3 expression and overall survival, HRs and 95% CIs were reported directly in 2 studies, and HRs and 95% CIs were extracted from the Kaplan-Meier survival curves in 5 studies. However, the method of extrapolating HR from survival curves may be less accurate than the direct method. Fourthly, the location of p-STAT3 expression was different in the included studies. In 12 studies, p-STAT3 was located in the nucleus, 1 study did not report the location, and in 4 studies, p-STAT3 was located in both the cytoplasm and nucleus. Finally, the number of samples, the different cut-off values, the fixation method for paraffin-embedded tissues, the regional sources of patients, age and follow-up time may be factors that influenced the pooled results.

In conclusion, our meta-analysis demonstrates that overexpression of p-STAT3 has a significant association with poorer overall survival and the presence of lymph node metastases in colorectal cancer patients. P-STAT3 is a potential predictor of poorer prognosis of colorectal cancer patients. To confirm our present results, more standardized studies are needed to be implemented in the future.

## Supporting Information

S1 Fig PRISMA Flow Diagram. (DOC)

S1 Table PRISMA Checklist. (DOC)
