# Supplementary material for: The Role of p-STAT3 as a Prognostic and Clinicopathological Marker in Colorectal Cancer: A Systematic Review and Meta-Analysis
Source: PLoS One. 2016 Aug 9;11(8):e0160125. doi: 10.1371/journal.pone.0160125 (PMC4978497; doi:10.1371/journal.pone.0160125)
Supplement: S1 Fig — (DOC) [file pone.0160125.s001.doc]

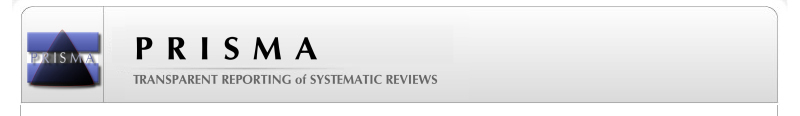
**PRISMA 2009 Flow Diagram**

**Screening**

**Included**

**Eligibility**

**Identification**

Records identified through database searching
(n = 1199 )

Additional records identified through other sources
(n = 0 )

Records after duplicates removed
(n = 1095 )

Records screened
(n = 1095 )

Records excluded
(n = 1027 )

Full-text articles assessed for eligibility
(n = 68 )

Full-text articles excluded, with reasons
(n = 51 )

Studies included in qualitative synthesis
(n = 17 )

Studies included in quantitative synthesis (meta-analysis)
(n = 17 )
